# Draft Genome Sequences of 13 Plant-Associated Actinobacteria of the Family *Microbacteriaceae*

**DOI:** 10.1128/MRA.00795-20

**Published:** 2020-09-17

**Authors:** Sergey V. Tarlachkov, Irina P. Starodumova, Lubov V. Dorofeeva, Natalia V. Prisyazhnaya, Semen A. Leyn, Jaime E. Zlamal, Sebastian Albu, Steven A. Nadler, Sergei A. Subbotin, Lyudmila I. Evtushenko

**Affiliations:** a All-Russian Collection of Microorganisms (VKM), G. K. Skryabin Institute of Biochemistry and Physiology of Microorganisms, Pushchino Scientific Center for Biological Research of the Russian Academy of Sciences, Pushchino, Russia; b Branch of Shemyakin and Ovchinnikov Institute of Bioorganic Chemistry, Russian Academy of Sciences, Pushchino, Russia; c Infectious and Inflammatory Disease Center, Sanford Burnham Prebys Medical Discovery Institute, La Jolla, California, USA; d A. A. Kharkevich Institute for Information Transmission Problems, Russian Academy of Sciences, Moscow, Russia; e California Department of Food and Agriculture, Sacramento, California, USA; f Department of Entomology and Nematology, University of California, Davis, California, USA; g Center of Parasitology of A. N. Severtsov Institute of Ecology and Evolution, Russian Academy of Sciences, Moscow, Russia; University of Arizona

## Abstract

Draft genome sequences of 13 bacterial strains from the family *Microbacteriaceae* were generated using Illumina technology. The genome sizes varied from 3.0 to 4.8 Mb, and the DNA G+C content was 68.1 to 72.5%. The sequences obtained will contribute to the development of genome-based taxonomy and understanding of molecular interactions between bacteria and plants.

## ANNOUNCEMENT

Members of the family *Microbacteriaceae* (class *Actinobacteria*) are widely distributed in various terrestrial and aquatic ecosystems and often occur in association with plants as endophytes and pathogens (1–3).

Novel strains of *Microbacteriaceae* were recovered from eight different plants of five families (Table 1) collected in various sites in California. *Rathayibacter* sp. strain VKM Ac-2835 was isolated from a *Malus* sp. with symptoms of bacterial wetwood disease by macerating several pieces of symptomatic superficial bark tissue in a sterile aqueous solution and then plating it onto *Pseudomonas* F agar (Becton, Dickinson, USA) amended with cycloheximide (100 mg/liter). The remaining strains were isolated from plants without visible symptoms of diseases, as described (3, 4), but Reasoner’s 2A (R2A) agar (Fluka Analytical, USA) was used as the plating medium for isolation. Rathayibacter agropyri CA-4^T^ (=VKM Ac-2828^T^) was kindly provided by T. D. Murray. For preservation, strains were grown on R2A agar and lyophilized using standard techniques. All strains were deposited in the All-Russian Collection of Microorganisms (VKM; http://www.vkm.ru).

**TABLE 1:** Characteristics and DDBJ/ENA/GenBank accession numbers of the genomes

Organism	Associated plant (family)	No. of reads	Coverage (×)	No. of scaffolds	Scaffold *N*_50_ (bp)	Genome size (Mbp)	G+C content (%)	No. of proteins	SRA accession no.	GenBank accession no.
*Rathayibacter agropyri* VKM Ac-2828^T^	Pascopyrum smithii (Poaceae)	14,362,336	578	25	656,029	3.0	68.1	2,835	SRX8466800	JABRPL000000000
*Rathayibacter* sp. VKM Ac-2835	Malus domestica (Rosaceae)	12,953,304	410	6	1,292,012	4.3	72.2	3,849	SRX8466801	JABSNQ000000000
*Rathayibacter* sp. VKM Ac-2857	Brachypodium distachyon (Poaceae)	21,681,636	692	8	1,409,124	4.6	72.1	4,080	SRX8466811	JABMLE000000000
*Rathayibacter* sp. VKM Ac-2856	*Brachypodium distachyon* (Poaceae)	20,040,560	689	9	783,576	4.3	72.5	3,806	SRX8466810	JABMLF000000000
*Rathayibacter* sp. VKM Ac-2858	*Brachypodium distachyon* (Poaceae)	22,149,976	762	9	783,576	4.3	72.5	3,806	SRX8466812	JABMLD000000000
*Curtobacterium* sp. VKM Ac-2852	Avena fatua (Poaceae)	19,012,470	724	7	1,020,126	3.9	70.8	3,580	SRX8466807	JABMLI000000000
*Curtobacterium* sp. VKM Ac-2861	*Marah* sp. (Cucurbitaceae)	17,600,848	642	15	624,972	4.0	70.8	3,739	SRX8466804	JABMLA000000000
*Frigoribacterium* sp. VKM Ac-2836	Fragaria vesca (Rosaceae)	28,426,948	1,100	10	1,120,753	3.3	70.4	3,010	SRX8466805	JABRPK000000000
*Frigoribacterium* sp. VKM Ac-2859	*Brachypodium distachyon* (Poaceae)	14,404,054	638	5	1,688,707	3.3	71.3	3,027	SRX8466802	JABMLC000000000
*Frigoribacterium* sp. VKM Ac-2860	*Brachypodium distachyon* (Poaceae)	14,248,970	632	5	1,688,688	3.3	71.3	3,029	SRX8466803	JABMLB000000000
*Herbiconiux* sp. VKM Ac-2851	Soliva sessilis (Asteraceae)	18,251,960	623	9	1,342,178	4.3	70.7	4,032	SRX8466806	JABMLJ000000000
*Microbacteriaceae* bacterium VKM Ac-2854	*Myosotis* sp. (Boraginaceae)	42,551,862	1,319	18	721,153	4.8	69.6	4,359	SRX8466808	JABMLH000000000
*Microbacteriaceae* bacterium VKM Ac-2855	*Myosotis* sp. (Boraginaceae)	17,323,178	542	26	386,786	4.7	68.3	4,255	SRX8466809	JABMLG000000000

Biomass for DNA extraction was grown in liquid peptone-yeast medium (5) inoculated with cells from a single colony, followed by cultivation at 28°C for 18 to 20 h on a rotary shaker. Genomic DNA was extracted using a QIAamp DNA minikit (Qiagen, Germany). DNA libraries for strains VKM Ac-2828^T^, VKM Ac-2835, and VKM Ac-2836 were prepared in-house using a NEBNext Ultra II FS DNA library prep kit for Illumina (New England Biolabs, USA) following the protocol for use with inputs of ≥100 ng with modifications as described previously (6). Pooled DNA libraries were sequenced by Novogene Co., Ltd., on an Illumina HiSeq X Ten instrument to obtain 150-bp paired-end reads. For the remaining strains, DNA library construction and sequencing were conducted by Novogene Co., Ltd. Libraries were generated using a NEBNext DNA library prep kit for Illumina (New England Biolabs) following the manufacturer’s recommendations. Pooled DNA libraries were sequenced on an Illumina NovaSeq 6000 instrument to obtain 150-bp paired-end reads.

Default parameters were used for all software unless otherwise specified. The quality of the reads was checked with FastQC 0.11.8 (7). Adapter sequences and low-quality regions in the raw reads were cut with Trimmomatic 0.39 (8) with the following options: ILLUMINACLIP:TruSeq3-PE-2.fa:2:30:10, SLIDINGWINDOW:4:15, and MINLEN:50. Trimmed reads were assembled using SPAdes 3.14.1 (9) with the following options: --cov-cutoff, auto; and --careful. The quality of assembly was assessed with QUAST 5.0.2 (10). Assemblies were annotated with NCBI PGAP (11) and the RAST Web server (12, 13). The pairwise similarity between the 16S rRNA gene sequences was determined using TaxonDC 1.3.1 (14). The average nucleotide identity (ANI) and digital DNA-DNA hybridization (dDDH) values were calculated using the JSpecies 1.2.1 (15) and GGDC 2.1 (16) tools, respectively.

**FIG 1:**
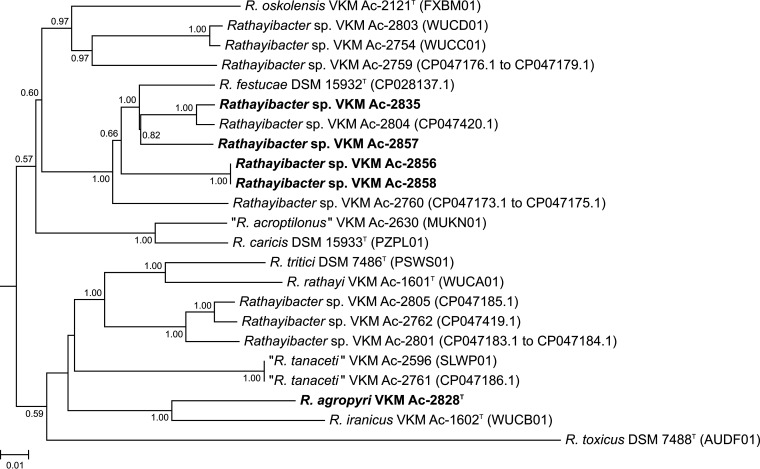
Phylogenomic tree based on genomes of *Rathayibacter* strains sequenced in this work (in bold) and members of other validly published and some putative (6, 19, 20) *Rathayibacter* species. The tree was inferred by the balanced minimum evolution method using JolyTree 1.1.181205ac (21) with branch lengths scaled to the estimated number of substitutions per site. Branch support values (rate of elementary quartets) above 0.5 are indicated at the branch points. The genomic sequence of Clavibacter michiganensis subsp. *sepedonicus* ATCC 33113^T^ (GenBank accession numbers AM849034.1 to AM849036.1) served as an outgroup (not shown) to root the tree.

Accession numbers and characteristics of the genomes are provided in Table 1. Figure 1 shows the phylogenomic positions of *Rathayibacter* strains sequenced here within the genus *Rathayibacter*. Four newly isolated strains clustered with Rathayibacter festucae but exhibited average nucleotide identity values (90.6 to 93.4%) and digital DNA-DNA hybridization levels (41.7 to 52.1%) to *R. festucae* DSM 15932^T^ not exceeding the thresholds for species delineation (17). No genome sequences of relevant type strains of the *Curtobacterium*, *Frigoribacterium*, and *Herbiconiux* species are available to precisely determine the phylogenomic positions of the remaining strains sequenced in this work (Table 1).

A BLAST search confirmed the presence of a genomic cluster comprising a complete suite of tunicaminyluracil-related biosynthetic genes in *R. agropyri* CA-4^T^ as already reported by Tancos et al. (18) for this strain. This gene cluster is not present in any other genomes sequenced in this work.

Further whole-genome sequencing of other *Microbacteriaceae* along with comparative genomic and phenotypic analyses of putative and known species with validly published names will result in valid descriptions of the revealed new taxa, contributing to the development of the genome-based taxonomy of prokaryotes.

### Data availability.

These whole-genome shotgun projects have been deposited in DDBJ/ENA/GenBank under the accession numbers listed in Table 1.
